# A systems perspective on early childhood development education in South Africa

**DOI:** 10.1186/s40723-022-00100-5

**Published:** 2022-06-29

**Authors:** Lieschen Venter

**Affiliations:** Department of Logistics, Stellenbosch University, Room 3026, Van der Sterr Building, Victoria Street, Stellenbosch, 7600 South Africa

**Keywords:** School readiness, System dynamics simulation modeling, Mathematical modeling, South African basic education system

## Abstract

South Africa’s basic education system is dysfunctional. It scores last or close to last in a myriad of metrics and delivers learners with some of the worst literacy and numeracy competencies worldwide. A bimodal distribution in the results exists when learners from the richest socioeconomic quintile are performing adequately well, while learners from the poorest quintiles are failing. This paper presents a system dynamics simulation model to describe the causal linkages between improved early childhood and pre-school learning practices on the education system as a whole. The paper investigates the difference in performance between rich and poor communities. Three interventions explore the research question of whether it is the number of enrolments into early childhood development programs that increases a cohort’s school readiness, or rather the quality of the early childhood development programs into which they were enrolled. The results answer the research question for the Western Cape province by showing that increasing the quality of the formal ECD programs leads to a greater percentage of school-ready five year olds than increasing the percentage of enrolled children, but that decreasing community poverty leads to better results than either intervention. The results show the simulation model to be a powerful tool to assist with policy setting and intervention testing for any other province or country by simply changing the input data and calibration.

## Introduction

The South African education system is in a precarious state. Since the abolishment of the 1953 Bantu Education Act which shaped the education system during the Apartheid era, significant inequalities still characterize the country in educational outcomes. Norling (2020) notes that the system still displays dysfunctional behavior despite the national government developing progressive policies that are in line with international trends. UNICEF (2020) released its 2020 Basic Education Budget Brief which highlighted that government policy on Early Childhood Development (ECD) had not been implemented effectively. ECD facilities across the country vary in quality and operations and in most cases relatives or non-profit organizations provide the service and it is not equitably accessible especially to those who need it most. The report emphasized the rising concern for the health of the education system stating that the repetition rates are increasing steadily from Grade 8 and peaking in Grade 10. Although the national government has made substantial progress to increase access to basic education the quality thereof is concerning. Being enrolled in school does not automatically translate to learning (World Bank, 2019).

The Trends in International Mathematics and Science Study (TIMSS) which records the Grade 4 and Grade 8 trends in mathematics and science was first conducted in 1995 and occurs every 4 years. TIMSS 2019 is, therefore, the seventh assessment. The Progress in International Reading Literacy Study (PIRLS) tests learners in Grade 4 in reading proficiency every 5 years and began in 2001. At a regional level, the Southern and Eastern Africa Consortium for Monitoring Educational Quality (SACMEQ) assesses Mathematics, reading English in Grade 6 learners. The first assessment (SACMEQ I) took place between 1995 and 1999 with the latest assessment (SACMEQ IV) being published in 2017.

The TIMSS revealed that out of 64 education systems that participated at the fourth grade, South Africa finished in 62nd place for both Mathematics and Physical Science (NCES, 2020). At the eighth grade, 46 systems participated and South Africa had the lowest score in Physical Science and achieved second to last place in Mathematics. Performance in languages was alarmingly low with 78% of Grade 4 learners unable to read with comprehension. The study also included other middle-income countries such as Iran scoring 35% and Chile with only 13% of fourth-graders that could not read for meaning (Mullis et al., 2017).

Along with its struggling education system, South Africa also suffers from one of the highest Gini indices in the world. The Gini index is a measure of statistical distribution intended to represent citizens of a country's income or wealth distribution and is usually used to indicate inequality. Bimodality in academic achievement occurs when historically disadvantaged schools are unable to produce quality education compared to more privileged schools. The presence of this bimodality indicates that while the South African Constitution promises equal access to education the country has not yet been able to provide equal quality of education. In the last two decades, the national government has tried to solve the education crisis by designating a larger portion of state funding towards the education system. In 2019 it allocated approximately 15% of the national budget to basic education expenditure (UNICEF, 2019). Despite the government’s best efforts to distribute resources this proves to be beneficial only to a certain extent. The majority of the system still fails to provide the quality of education needed for sustainable growth.

South African babies and toddlers attend early childhood development programs within this context of dysfunction and inequality. *Early childhood development* (ECD) refers to the physical, socio-emotional, cognitive, and motor development that a child experiences between birth and the age of eight. The early years are important as this is the period when the brain develops the quickest and has a high capacity for change. According to the South African Early Childhood Review for 2019 (Hall et al., 2019), of the 3.1 million children in South Africa between the ages of 3 and 5 years, 69% were attending a group program. These group programs include playgroups, community-based programs, nursery schools, and Grade R.

Economic inequality in South Africa affects the ability of parents to provide their children with quality ECD education. The 2011 national census revealed that 44.5% of the households in South Africa qualified as low income and no income households and 48.3% qualified as middle income (Statistics South Africa, 2012). This means that a large portion of children belong to a lower socio-economic class and cannot afford structured ECD programs. These children will not attend group programs but instead, have an informal ECD experience such as being taken care of by a day mother or a relative. These caregivers have often not received the training required to run quality ECD programs. In fact, according to a 2013 audit, only 10% of early childhood educators had any qualification higher than Grade 12 (Kotzé, 2015).

Some interventions could lead to the successful implementation of proper structures in ECD programs. The national government drafted an action plan in 2015 with 27 goals for the realization of an improved education system by 2030. Goal 11 is to improve the access of children under the age of six to quality ECD education below Grade 1 (South Africa, Department of Basic Education, 2015).

Grade R, or reception year, is an optional year of instruction before enrolment into the compulsory Grade 1. During this grade, learners encounter structured learning for the first time with a formal curriculum focused on language, mathematics, and life skills. Van der Berg et al. (2013) looked at the DBE’s *Annual National Assessment* (ANA) results from 2012 and found that children who had been exposed to Grade R had increased mathematics and home language scores in later school years. The increased scores were higher in the more affluent communities, but often not measurable in the poorer communities. From this, they recognized that quality ECD education and Grade R learning are essential in preparing for foundation phase education.

Kotzé (2015) analyzed the likelihood of successful implementation of the National Development Plan. The plan proposed universal accessibility to two years of ECD education before Grade R. She found that participation rates in ECD programs had increased significantly. She showed an increase in participation rates in all the age groups with the largest increase of 38% among the four year olds. In 2013 64% of four year olds, and 81% of five year olds in South Africa attended an education institution (Kotzé, 2015). However, a large majority of the facilities were inadequate, very few centers had adequate teaching materials, and barely any of the teachers at any of the centers were adequately trained. From this Kotzé concluded that the potential impact of pre-Grade R on subsequent learning is high. However, the quality of many ECD programs needed to be improved to reach this potential.

An ECD program provides services to children and caregivers to promote school readiness. Programs are structured within ECD facilities to provide learning and support appropriate for a child’s developmental age and stage. Programs are offered formally at ECD centers, and child-and-youth centers, or informally as non-center-based programs.

Various forms of non-center-based programs exist. Home-based programs at the household level are offered to primary caregivers, and young children to support early stimulation and development. Home-based programs also promote referrals and linkages to support services. Community-based programs are provided at community structures, such as clinics, schools, traditional authority offices, municipal offices, community halls, or churches. These programs are provided by trained community members and may operate two or three days per week. Mobile programs are offered to children in rural and farming areas. Playgroups can be organized for young children to promote learning and play. Toy libraries provide children and families with access to developmentally appropriate educational play and learning materials. Finally, childminding is a program for a maximum of six children in the care of a person during the day as arranged by the primary caregiver.

Goal 11 of the Action Plan to 2030 is to improve the access of children under the age of six to quality ECD education below Grade 1 (South Africa, Department of Basic Education, 2015). The lack of consideration of the quality of these programs, and especially of informal programs, could cause the only outcome to simply be increased enrolments without increasing cognitive development and school readiness. A system dynamics (SD) model of the complexity of early life as a South African child enables analysis of the efficacy of a system with increase enrolment rates.

Richmond (1993) described the complexity of the systems within which humans observe the planet's problems as growing faster than our ability to understand it. Traditional solutions no longer work as the subsystems in a finite earthly habitat grow across and into each other with unpredictable and poorly understood causality. We are too often caught off-guard by the counter-intuitive implications of the simple solutions we used to employ. Richmond suggests systems thinking, and SD modelling, as the solution for analysing and solving our most complex problems.

SD is the simulation of systems thinking, where non-linear, first-order differential, and integral equations are used to model the flow of data between a system’s components. It is used to model aggregate values instead of an individual entity’s characteristics as is the case in, for example, agent-based simulation modeling. This enables the modeler to discover the endogenous causes driving a system. Exogenous stimuli may pulse input into a system, but changes over time occur predominantly through internal feedback. Education systems are naturally complex, interconnected, and policy-laden systems and their designs are unique and challenging to conceptualize. SD is a recommended and intuitive technique one can use to investigate and analyze the internal dynamics of education systems.

SD simulation at the correct level of abstraction and complexity opens up the “black box” of school functionality and efficiency. By simulating each part of the system, the complex relationships can be studied and manipulated so that the connection between resources invested and outcomes achieved may be better understood.

This paper presents the *Early Childhood Development Model* (ECDM) to describe the causal linkages between improved ECD and Grade R learning practices on the education system as a whole. The ECDM is constructed using the system dynamics simulation methodology. It is the best approach to understanding the nonlinear behavior of the complex ECD systems time using stocks, flows, internal feedback loops, table functions, and time delays. The paper gives insight into the mechanics of the ECDM's design, followed by a brief discussion on the input data and assumptions used to populate the model. The system status quo is presented as base case results, followed by interventions and a discussion of results. The paper finishes with some concluding observations on the results. The results answer the research question to whether it is the number of enrolments into ECD programs that increases a cohort's readiness for primary school education, whether it is the quality of the ECD programs into which they were enrolled that increases readiness, or whether an intervention on some other variable all together leads to the best solution for the system.

## Literature

The ECDM simulates the progression of children from birth to their enrolment into primary school at Grade R or Grade 1. Nine factors identified from literature impacts this progression: Poverty, the health of the children, their level of stunting, family support, infrastructure, program quality, ECD practitioner quality, the aptitude of the children, and their later academic success which in turn affects their progeny.

Poverty is determined by a person's access to income, employment, basic services, ownership of assets, social inclusion, and participation in decision-making. Absolute poverty describes an average monthly income that is less than the absolute minimum required before the earner has to decide between procuring food and important non-food items. Relative poverty describes an income that is less than what others in society are earning. Subjective poverty describes an income that is unable to meet the household’s needs. Absolute poverty is the best measure for developing countries such as South Africa as approximately 17 million people (or about a third of the country) live at this level. South African households can be classified according to their socio-economic quintile by monthly income. The lowest three quintiles earn below ZAR2 340 and fall under the 2018 living monthly wage level of ZAR6 460 with 22% of the population falling under the food poverty line of ZAR335 (i.e., their income is insufficient to purchase 2 100 cal per day, the minimum daily requirement in an emergency). The fourth quintile has an average monthly income between ZAR2 341 and ZAR5 956. In the richest quintile, 10% of all South Africans earn more than ZAR7 313, 5% earn more than ZAR11 091, and 2% earn more than ZAR19 089 per month (Ruch, 2014).

Increasing poverty has a decreasing effect on the quality of childhood health. Health is a measure of the physical well-being of the children in a community. Good health results from access to clean, warm homes with adequate ventilation, quality healthcare in the form of doctors, hospitals, clinics, and pharmacies, proper sanitation practices, food security, clean water, and regular refuse removal. Children from poor households suffer worse health and die younger than the rich. They have higher than the average child and maternal mortality, higher levels of disease, and limited access to quality health care and good nutrition (World Health Organisation, 2013).

Decreasing health increases stunting. Stunting occurs when a child is prevented from growing physically, or developing cognitively due to poor nutrition, repeated illness, and a lack of psychosocial interaction. As a result, they fall behind their peers. A low height-for-age is the best indicator of the presence of childhood stunting (Casale et al., 2014). Casale and Desmond (2016) applied a multivariate regression analysis to a data set of children born in urban South Africa in 1990 and found that poor child health, particularly poor nutrition, results in stunting. In previous work, Casale et al. (2014) found a large and significant association between stunting of two year olds and their cognitive function at the age of five years. Early childhood stunting is negatively associated with the cognitive development of children and, therefore, lowers their cognitive function, or aptitude (Dewey & Begum, 2011).

Aptitude refers to a child’s developmental progress at each stage between birth and the age of five according to the *Revised Denver Pre-screening Developmental Questionnaire* (R-DPDQ) (Frankenburg et al., 1987). The questionnaire evaluates a child's development in the areas of personal and social skills, fine and gross motor skills, language, and problem-solving ability. Table 1 contains, for example, the physical, cognitive skills that a five year old should achieve. A child who has achieved these may be deemed ready for formal education.

**Table 1:** R-DPDQ required physical, communication, and cognitive skills for five year old children

Number	Description
1	The child can dress themselves without help
2	The child can play any simple board or card game
3	The child can brush their teeth without help or supervision
4	The child can prepare a bowl of cereal without making too much of a mess
5	The child can build a tower of blocks
6	The child can count the number of blocks
7	The child can imitate a vertical line
8	The child can copy a circle
9	The child can copy a cross
10	The child can copy a square
11	The child can identify which line is longer
12	The child can draw a person using three parts
13	The child can draw a person using six parts
14	The child understands prepositions
15	The child can name colors
16	The child can identify words
17	The child knows adjectives
18	The child knows opposites
19	The child can wiggle their thumbs
20	The child can balance on one foot
21	The child can hop on one foot
22	The child walks heel-to-toe
23	The child’s speech is understandable

Increasing poverty has a decreasing effect on family support. Family support refers to the level of financial, emotional, mental, and physical support that children receive from family members. It can be measured by the number of parents present in the household, the level of parental education, the level of parental involvement, the number of learning-related resources, and the number of assets in the home (Visser & Juan, 2015). Children may find themselves growing up in a nuclear family (where spouses or partners couple with their children and no other members), a lone parent family (where a single parent has their children and no other members), an extended family (that is not a nuclear or lone parent family, but all members are related), or a composite family (that is not a nuclear or lone parent family, and some members are not related). A South African child qualifies for a governmental *Child Support Grant* (CSG) if their primary caregiver earns less than ZAR3 300 per month. Approximately 12.4 million children qualified during 2019, and of these, only about 25% of children came from families, where both parents were present (South Africa, Department of Planning, Monitoring and Evaluation, 2017). Parents from poorer communities are, therefore, less able to provide the presence and support necessary to stimulate early childhood development. The lack of stimulation and support from a struggling family increases a child's stunting (Duncan et al., 2014).

Poverty decreases the access of a community’s ECD practitioners and the adequate training they require. A certificate in Early Childhood Development is the absolute minimum childcare qualification that a practitioner requires to be considered a quality childcare provider or early childhood educator (September, 2009). Practitioner quality describes the overall rating of the ECD practitioners in the community. The level of education received by the practitioner, the tools achieved during ECD-specific training, the level of practitioner absenteeism, and the class size that a practitioner is assigned determines the quality of a practitioner. Class size can either enable or prohibit practitioners from giving children individual attention. Increasing practitioner quality increases the quality of the ECD programs. A quality program is one where children learn the correct skill at the correct time, in the correct way, so that an increase in quality has a positive effect on aptitude. The greatest gain in cognitive ability comes from participation in programs for two years or more at a minimum of 15 h per week (preferably at 30 h per week), where children are enrolled before the age of four (Loeb et al., 2007).

Finally, increasing poverty has a decreasing effect on infrastructure. Infrastructure refers to the presence of learning materials, sufficient facilities, and additional resources at each ECD center. ECD centers in poorer communities are often without electricity, roads, water, and an adequate supply of books and toys. The improvement of infrastructure has a positive effect on ECD quality.

**Fig. 1:**
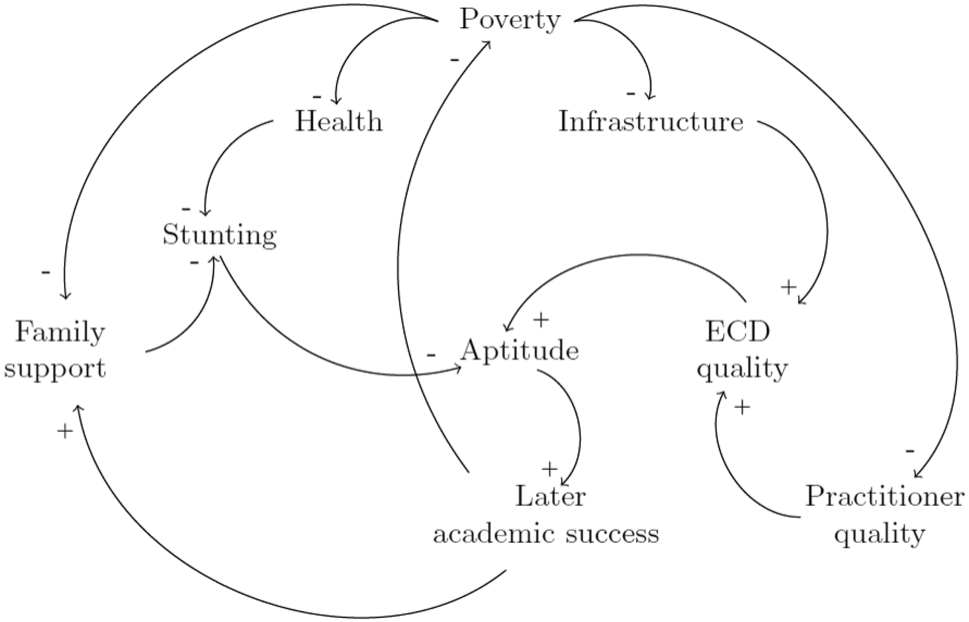
Causal loop diagram for early childhood development

Figure 1 contains the causal relationships between each of the factors within the early childhood system. Four main reinforcing loops drive the early childhood system.

The General Household Survey (GHS) (Statistics South Africa, 2016) remains the main data set for quantifying the various factors of the early childhood system by socio-economic class. Each year in the Western Cape (WC), the parents of about 700 children below the age of six are surveyed about multiple aspects of their children’s development and living situation. This data are supported by the five waves of the *National Income Dynamics Survey* (NIDS), where about 500 children are surveyed each year (Southern Africa Labour & Development Research Unit, 2018). The normalization of survey responses and test results ensures that factor scores can be brought into relationship with each other without unit discrepancies. Response options for survey questions are ranked from most to least desirable state and goodness scores are assigned to each response option in ascending order. The least desirable state is assigned a goodness score of 0 and the most desirable state is assigned a goodness score of 1. Intermediary states are assigned goodness scores linearly between these two extremes. The sum-product of the number of responses per response option and its goodness score provides the initial value for each factor.

Table 2 contains the average number of WC newborns to fiver year olds per quintile and per family type from 2010 to 2017. The nuclear family is the ideal structure to provide family support (Akomolafe & Olorunfemi-Olabisi, 2011; Ella et al., 2015) and it is, therefore, assigned a score of 1. Children from lone families experience greater support as at least one of their biological parents are present within the household and the family type is assigned a higher score of 0.6 when compared to the score of 0.3 for extended families. Children from composite families are the worst off and the family type is, therefore, assigned a score of 0.

**Table 2:** Average percentage of children per socio-economic quintile in each of the four family types

Family type	Goodness score	Quintiles 1–3	Quintiles 4 and 5
Composite	0.0	3%	1%
Extended	0.3	13%	12%
Lone	0.6	48%	29%
Nuclear	1.0	36%	58%
*F*^*F*^ initial value		0.69	0.79

Table 3 contains the average household monthly income for WC children under the age of six per quintile from 2010 to 2017. The lowest income bracket describes utmost poverty and is, therefore, assigned a goodness score of 0. The highest income bracket is assigned a goodness score of 1 and the remaining brackets are scored evenly between these extremes. The large difference between the scores for each quintile is an accurate mirror of South Africa's high Gini index.

**Table 3:** Average percentage of children per socio-economic quintile per monthly household income level

Monthly household income	Goodness score	Quintiles 1–3	Quintiles 4 and 5
Below R540	0.0	28%	0%
R541 to R1 151	0.3	19%	0%
R1 152 to R2 340	0.5	53%	0%
R2 341 to R5 965	0.8	0%	51%
Over R5 966	1.0	0%	49%
*F*^*V*^ initial value		0.32	0.90

The GHS of 2016 is the first version to survey the quality of ECD facilities and infrastructure. Table 4 contains the percentage of WC children below the age of six who attend facilities, where the listed infrastructure was present. There is little difference between the facilities in each quintile and the averages of these percentages become the ECDM score.

**Table 4:** Percentage of children per socio-economic quintile, where the necessary infrastructure is present

Infrastructure element	Quintiles 1–3	Quintiles 4 and 5
Pictures on the walls	92%	92%
Tapped or piped water	94%	95%
Electricity	96%	97%
Flushing toilets	89%	95%
Educational toys	88%	90%
Fenced facility	84%	90%
Outside play area	92%	92%
Outside play equipment	76%	87%
*F*^*I*^ initial value	0.89	0.92

Table 5 contains the percentage of WC children below the age of six per health category. There is surprisingly little difference between the general health of children in each quintile. This similarity may be due to the subjective nature of classifying health, where richer communities might have a higher expectation of what constitutes good health.

**Table 5:** Average percentage of children per socio-economic quintile by health status

Health status	Goodness score	Quintiles 1–3	Quintiles 4 and 5
Poor	0.0	0%	0%
Fair	0.3	1%	0%
Good	0.5	12%	14%
Very good	0.8	44%	36%
Excellent	1.0	43%	49%
*F*^*H*^ initial value		0.85	0.85

Again, the GHS of 2016 is the first version to survey the level of stimulation children receive from their ECD practitioner. Tables 6 and 7 contain the percentage of WC children below the age of six who receive different forms of cognitive stimulation. It is assumed that these three stimulation techniques play an equal role in the cognitive development of a child. Consequently, the averages of these percentages become the initial value for practitioner quality.

**Table 6:** Percentage of children receiving cognitive stimulation in Quintiles 1–3

Activity frequency	Goodness score	Talking	Storytelling	Singing	Average
Never	0.0	8%	28%	12%	16%
Sometimes	0.3	19%	27%	32%	26%
Often	0.6	19%	18%	21%	19%
Always	1.0	54%	28%	35%	39%
*F*^*Y*^ initial value	0.58

**Table 7:** Percentage of children receiving cognitive stimulation in Quintiles 4 and 5

Activity frequency	Goodness score	Talking	Storytelling	Singing	Average
Never	0.0	15%	21%	9%	15%
Sometimes	0.3	7%	17%	24%	14%
Often	0.6	16%	17%	24%	19%
Always	1.0	62%	46%	49%	53%
*F*^*Y*^ initial value	0.68				

Table 8 contains the average parental educational level for WC children under the age of six per quintile from 2010 to 2016. Parental educational level is an important predictor of children’s later academic success as it determines the level of academic support parents can provide and the level of future academic aspirations they set for their children (Davis-Kean, 2015; Dubow et al., 2010). The highest level of parental education is a qualification from a tertiary education institution and we assign a goodness score of 1 to this category. Parents who completed only high school are not ideal, but still able to provide better support than parents who completed only primary school (Asad et al., 2015; Campbell et al., 2000; Thompson et al., 1988). A goodness score of 0.6 and 0.3 is assigned to these, respectively. Parents with no formal education provide the least support and have a goodness score of 0. The household head is self-defined by the household and used simply as a construct to determine individuals’ relational status to each other. No guidance is given that the household head must be the eldest, highest earner, or of a specific gender.

**Table 8:** Average percentage of children per socio-economic quintile per education level

Parental educational level	Goodness score	Quintiles 1–3	Quintiles 4 and 5
None or informal	0.0	1%	1%
Primary	0.3	20%	6%
Secondary	0.6	67%	54%
Tertiary	1.0	12%	39%
*F*^*L*^ initial value		0.58	0.73

The NIDS surveys the height of children below the age of six. A child is considered severely stunted if their height-for-age is three standard deviations below the mean of a healthy reference population set by the World Health Organization (2013). A child is considered stunted if their height is two standard deviations below this reference. Table 9 contains the average percentage of WC children under the age of six per quintile at each level of stunting from 2010 to 2017. Severely stunted children are at the greatest disadvantage and are assigned a goodness score of 0. Children who experience no stunting are best off and are assigned a goodness score of 1 with stunted children having a goodness score of 0.5.

**Table 9:** Average percentage of children at each level of stunting per socio-economic quintile

Level of stunting	Goodness score	Quintiles 1–3	Quintiles 4 and 5
Severely stunted	0.0	22%	22%
Stunted	0.5	17%	11%
Not stunted	1.0	52%	67%
*F*^*T*^ initial value		0.60	0.73

The GHS does not contain a survey to record the cognitive skills of children below the age of five. The Early Learning Outcomes Measure (ELOM) is the first program to measure the performance of South African children aged 50–59 months and 60–69 months, respectively. It includes 23 items measuring indicators of a child's early development in five domains: gross motor development, fine motor coordination and visual–motor integration, emergent numeracy and mathematics, cognition and executive functioning, and emergent literacy and language as listed in Table 1 (Dawes et al., 2016). Because no data set yet exists for children below this age, the level of aptitude at age five is used as a continuation of the level of aptitude of all ages below and assign the same score to all children aged five and younger. Table 10 contains the percentage of WC children aged 50–69 months at each achievement level during 2016.

**Table 10:** Average percentage of children at each level of aptitude per socio-economic quintile

Level of performance	Goodness score	Quintiles 1–3	Quintiles 4 and 5
At-risk	0.0	29%	16%
Falling behind	0.5	24%	34%
Achieving the standard	1.0	46%	49%
*F*^*B*^ initial value		0.58	0.66

As an ECD program consists of practitioners enabled by their infrastructure, the initial value for ECD quality may be taken as the average initial values of infrastructure and practitioner quality.

An average of 60% of children under the age of two in the Western Cape are enrolled into ECD programs from 2010 to 2016 (Southern Africa Labour & Development Research Unit, 2018). It is difficult to interpret this data for meaning, because it is impossible to determine what the respondents to the survey regard as appropriate education for children of this age. Better data are needed for this age group. However, Table 11 contains the average percentage of WC children enrolled in formal programs. Table 12 contains the average percentage of stunted children by age in years, socio-economic quintile, and ECD enrolment status in the Western Cape from 2014 to 2017 (Southern Africa Labour & Development Research Unit, 2018). These values initialize the stocks simulating early childhood progression. The stock-and-flow diagram in Fig. 3 depicts this process.

**Table 11:** Average percentage of children enrolled in ECD programs

Age	Quintiles 1–3		Quintiles 4 and 5	
	Enrolled	Not enrolled	Enrolled	Not enrolled
0	56%	44%	60%	40%
1	68%	44%	69%	31%
2	65%	35%	70%	30%
3	71%	29%	77%	23%
4	77%	23%	82%	18%
5	97%	3%	98%	2%

**Table 12:** Average percentage of children classified as stunted by age in years, socio-economic quintile, and ECD enrolment status

Age	Stunting	Quintiles 1–3		Quintiles 4 and 5	
		Enrolled	Not enrolled	Enrolled	Not enrolled
0	Severely stunted	0%	47%	0%	0%
	Stunted	0%	0%	0%	3%
	Not stunted	100%	53%	100%	43%
1	Severely stunted	15%	47%	17%	49%
	Stunted	5%	15%	20%	15%
	Not stunted	80%	37%	63%	36%
2	Severely stunted	25%	45%	8%	13%
	Stunted	25%	11%	0%	17%
	Not stunted	50%	44%	92%	71%
3	Severely stunted	14%	26%	14%	14%
	Stunted	12%	20%	13%	47%
	Not stunted	74%	55%	72%	39%
4	Severely stunted	10%	8%	8%	2%
	Stunted	24%	34%	8%	6%
	Not stunted	67%	58%	84%	92%
5	Severely stunted	4%	5%	2%	7%
	Stunted	26%	36%	2%	7%
	Not stunted	70%	59%	96%	86%

Some children spend the first five years of their lives enrolled in ECD education. Those who don't may enroll at a later stage as they grow older, or remain outside of the system until they have to enroll in primary school for Grade 1 at the age of six. The ECDM assumes children never leave the ECD system once they've entered into it. The children exhibit a level of age-appropriate cognitive readiness assumed to be equal to their level of stunting. The ECDM assumes stunted or severely stunted children are not ready to perform the tasks expected of them at each age. For children outside of the system, their readiness remains unchanged from its initial formation. For children within the system, their readiness can be improved upon or lost depending on the strength of the system. This strength is quantified as the resultant support for child aptitude from the interaction between the elements in Fig. 1.

## Methods

**Fig. 2:**
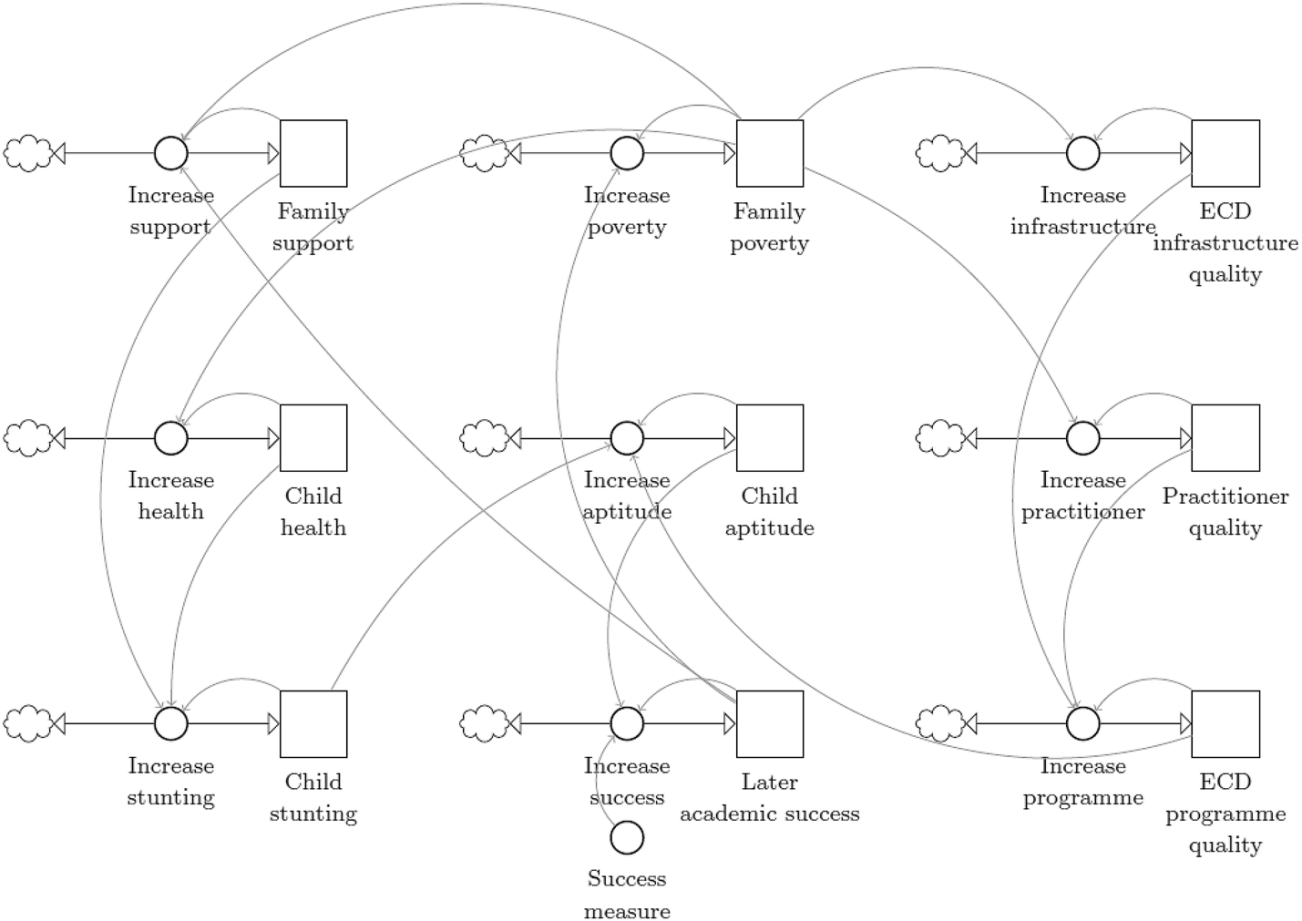
Stock-and-flow diagram for the early childhood system in the ECDM

Figure 2 contains the stock-and-flow diagram for the early childhood system. Each factor increases or decreases the other through bi-flows to achieve a new total aptitude score for each simulation time step. This aptitude score then serves as the input into child progression trains.

Let *F*^*F*^ be the score for family support, *F*^*V*^ be the score for family poverty, *F*^*I*^ be the score for ECD infrastructure quality, *F*^*H*^ be the score for child health, *F*^*B*^ be the score for child aptitude, *F*^*Y*^ be the score for practitioner quality, *F*^*T*^ be the score for child stunting, *F*^*L*^ be the score for later academic success, and *F*^*D*^ is the score for ECD program quality.

The change in each factor during the simulation period is described mathematically by

$$\frac{{\mathrm{d}F}^{F}}{\mathrm{d}t}= \frac{{F}^{F}+{F}^{P}+{F}^{L}}{3}-{F}^{F},$$

$$\frac{{\mathrm{d}F}^{V}}{\mathrm{d}t}= \frac{{F}^{V}+{F}^{L}}{2}-{F}^{V},$$

$$\frac{{\mathrm{d}F}^{V}}{\mathrm{d}t}= \frac{{F}^{I}+{F}^{P}}{2}-{F}^{I},$$

$$\frac{{\mathrm{d}F}^{H}}{\mathrm{d}t}= \frac{{F}^{H}+{F}^{P}}{2}-{F}^{H},$$

$$\frac{{\mathrm{d}F}^{B}}{\mathrm{d}t}= \frac{{{w}^{B}F}^{B}+{{w}^{T}F}^{T}+{w}^{D}{F}^{D}}{{w}^{B}+{w}^{T}+{w}^{D}}-{F}^{B},$$

$$\frac{{\mathrm{d}F}^{Y}}{\mathrm{d}t}= \frac{{F}^{Y}+{F}^{P}}{2}-{F}^{Y},$$

$$\frac{{\mathrm{d}F}^{T}}{\mathrm{d}t}= \frac{{F}^{T}+{F}^{H}+{F}^{F}}{3}-{F}^{T},$$

$$\frac{{\mathrm{d}F}^{L}}{\mathrm{d}t}= \frac{{F}^{L}+{F}^{B}}{2}-{F}^{L}, {\text{and}}$$

$$\frac{{\mathrm{d}F}^{D}}{\mathrm{d}t}= \frac{{F}^{D}+{F}^{P}+{F}^{Y}}{3}-{F}^{D}$$

where *w*^*B*^, *w*^*T*^, and *w*^*D*^ are the weighted influence of child aptitude, child stunting, and ECD quality, respectively, on child aptitude, the factor which influences progression at each age. Calibrating weights for all the terms in these equations would result in an infeasible 10^22^ simulation runs for each 0.1 increment between 0 and 1. Simplified weight calibration for each term at values of 0, 0.5, and 1 each would not only be too blunt but still result in an infeasible number of 3^22^ simulation runs. Therefore, a feasible 10^3^ simulation runs are used to calibrate the three weights directly impacting the output variable, *F*^*B*^. This output variable is received by the child progression chains.

Surveys and test results inform the initial values for the factors and are listed in Tables 2, 3, 4, 5, 6, 7, 8, 9, 10, 11, and 12. Table 13 contains a summary of the initial values for the ECDM factor scores, where a score closer to a maximum of 1 is a stronger factor achievement.

**Table 13:** Initial values for the factor scores of the ECDM

ECDM factor	Symbol	Quintiles 1–3	Quintiles 4 and 5
Child aptitude	*F*^*B*^	0.58	0.66
ECD program quality	*F*^*D*^	0.74	0.80
Family support	*F*^*F*^	0.75	0.79
Practitioner quality	*F*^*Y*^	0.58	0.68
Child health	*F*^*H*^	0.85	0.85
ECD infrastructure quality	*F*^*I*^	0.89	0.92
Later academic success	*F*^*L*^	0.58	0.73
Family poverty	*F*^*V*^	0.32	0.90
Child stunting	*F*^*T*^	0.60	0.73

**Fig. 3:**
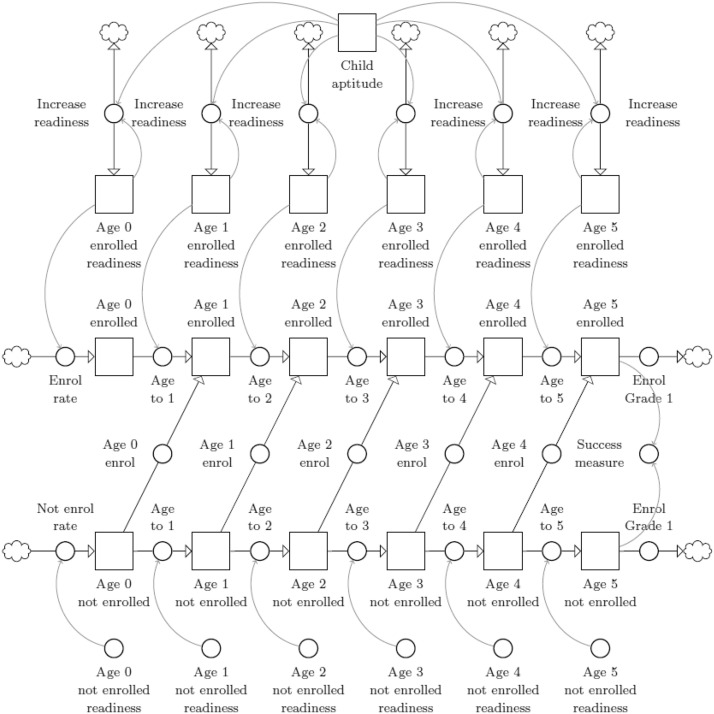
Stock-and-flow diagram for child progression in the ECDM

Figure 3 contains the stock-and-flow diagram of the simulation for child progression in the ECDM. Let ${F}_{j}^{b}$ be the child aptitude or readiness for children of age (in years) $j \in \left[\mathrm{0,1},\mathrm{2,3},\mathrm{4,5}\right]$ enrolled in formal ECD programs. During each iteration, the change in readiness depends on the strength of the early childhood system so that

$$\frac{{\mathrm{d}F}_{j}^{b}}{\mathrm{d}t}=\frac{{F}_{j}^{b}+ {F}^{B}}{2}$$

for all *j*. Similarly, let ${\tilde{F }}_{j}^{b}$ be the child aptitude or readiness for children of age (in years) $j \in \left[\mathrm{0,1},\mathrm{2,3},\mathrm{4,5}\right]$ not enrolled in formal ECD programs.

Let ${{\varvec{C}}}_{j}$ be the percentage of children of age (in years) *j* who are enrolled into formal ECD programs and who are either cognitively ready (*r*) for that age, or not ready ($\tilde{r }$), respectively, so that

$${{\varvec{C}}}_{j}=\left[{C}_{j}^{r},{C}_{j}^{\tilde{r }}\right]$$

where $j \in \left[\mathrm{0,1},\mathrm{2,3},\mathrm{4,5}\right]$. Similarly, let ${\stackrel{\sim }{{\varvec{C}}}}_{j}$ be the percentage of children who are not enrolled in formal ECD programs so that

$${\stackrel{\sim }{{\varvec{C}}}}_{j}=\left[{\tilde{C }}_{j}^{r},{\tilde{C }}_{j}^{\tilde{r }}\right]$$

for all *j*. Let $\beta$ be the percentage of babies enrolled in formal ECD programs, let ${\lambda }_{j}$ be the percentage of children of age $j \in \left[\mathrm{0,1},\mathrm{2,3},\mathrm{4,5}\right]$ that enroll for ECD education and recall ***E***_1_ as the enrolment into Grade 1 so that

$$\frac{{{\text{d}}C_{j} }}{{{\text{d}}t}} = \left\{ {\begin{array}{*{20}l} {\left[ {\beta F_{0}^{B} - C_{1}^{r} ,\beta \left( {1 - F_{0}^{B} } \right) - C_{1}^{{\tilde{r}}} } \right]} & {{\text{for}}\,{\mkern 1mu} j = 0,} \\ {\left[ {F_{j}^{B} C_{{j - 1}}^{r} - \lambda _{{j - 1}}^{r} - F_{{j + 1}}^{B} C_{j}^{r} ,\left( {1 - F_{j}^{B} } \right)C_{{j - 1}}^{{\tilde{r}}} - \lambda _{{j - 1}}^{{\tilde{r}}} - \left( {1 - F_{{j + 1}}^{B} } \right)C_{j}^{{\tilde{r}}} } \right]} & {{\text{for}}\,{\mkern 1mu} j\varepsilon \left[ {1,2,3,4} \right],{\text{and}}} \\ {\left[ {F_{j}^{B} C_{{j - 1}}^{r} + \lambda _{{j - 1}}^{r} ,\left( {1 - F_{j}^{B} } \right)C_{{j - 1}}^{{\tilde{r}}} + \lambda _{{j - 1}}^{{\tilde{r}}} } \right] - \varvec{E}_{1} } & {{\text{for}}\,{\mkern 1mu} j = 5} \\ \end{array} } \right.$$

and similarly,

$$\frac{{{\text{d}}C_{j} }}{{{\text{d}}t}} = \left\{ {\begin{array}{*{20}l} {\left[ {\left( {1 - \beta } \right)\tilde{F}_{0}^{B} - \tilde{C}_{1}^{r} ,\left( {1 - \beta } \right)\left( {1 - \tilde{F}_{0}^{B} } \right) - \tilde{C}_{1}^{{\tilde{r}}} } \right]} & {{\text{for}}\,j = 0,} \\ {\left[ {\tilde{F}_{j}^{B} \tilde{C}_{{j - 1}}^{r} - \lambda _{{j - 1}}^{r} - \tilde{F}_{{j + 1}}^{B} \tilde{C}_{j}^{r} ,\left( {1 - \tilde{F}_{j}^{B} } \right)C_{{j - 1}}^{{\tilde{r}}} - \lambda _{{j - 1}}^{{\tilde{r}}} - \left( {1 - \tilde{F}_{{j + 1}}^{B} } \right)\tilde{C}_{j}^{{\tilde{r}}} } \right]} & {{\text{for}}\,{\kern 1pt} j\varepsilon \left[ {1,2,3,4} \right],{\text{and}}} \\ {\left[ {\tilde{F}_{j}^{B} \tilde{C}_{{j - 1}}^{r} + \lambda _{{j - 1}}^{r} ,\left( {1 - \tilde{F}_{j}^{B} } \right)\tilde{C}_{{j - 1}}^{{\tilde{r}}} + \lambda _{{j - 1}}^{{\tilde{r}}} } \right] - E_{1} } & {{\text{for}}\,{\kern 1pt} j = 5.} \\ \end{array} } \right.$$

### Validation and sensitivity analysis

**Fig. 4:**
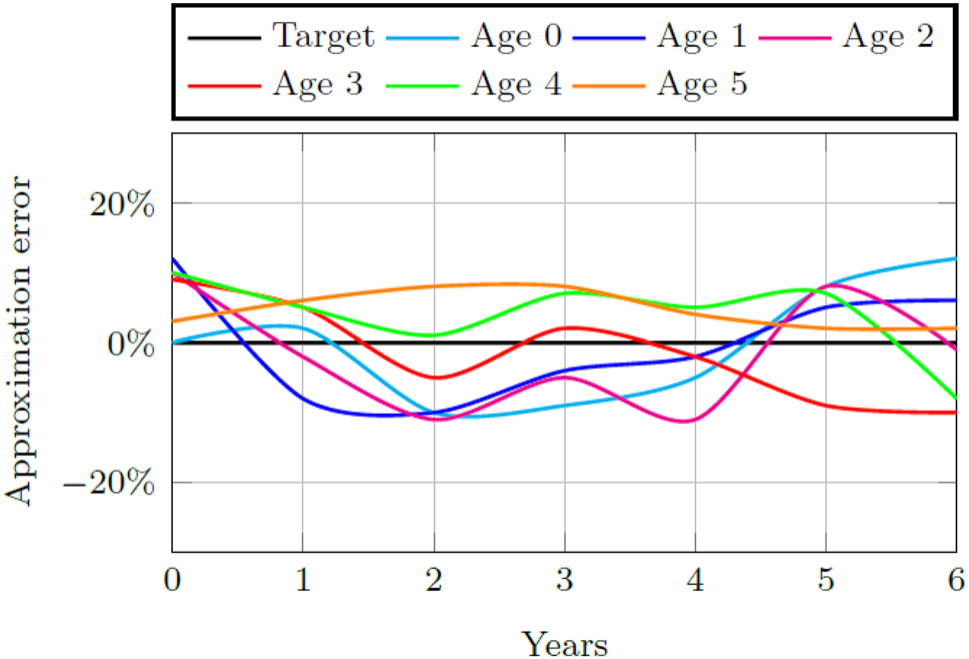
Model approximation of reality for the calibrated weights for the number of ECD enrolled children per age group in Quintiles 1–3

**Fig. 5:**
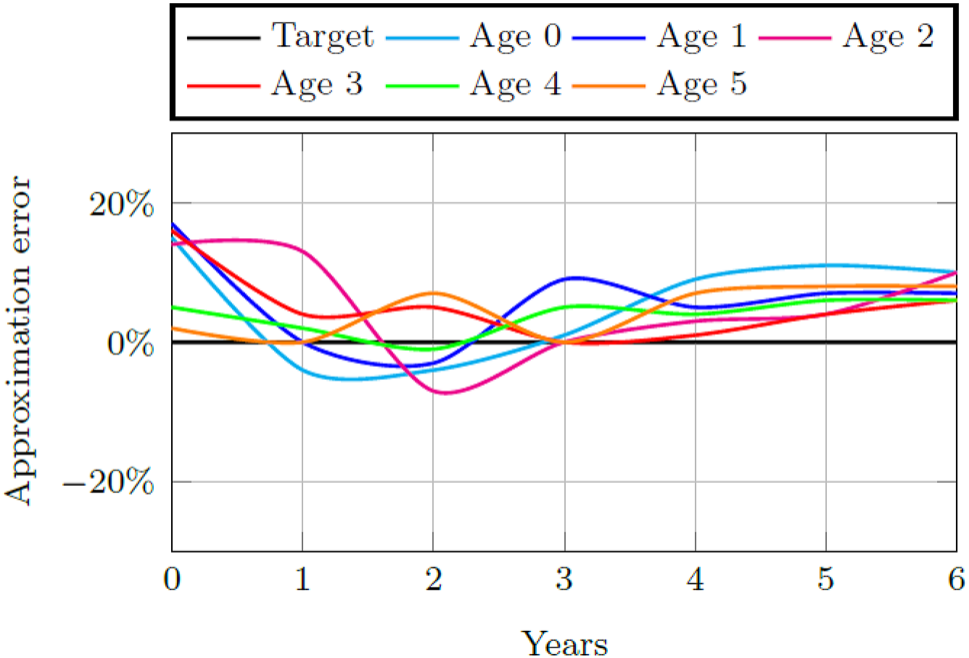
Model approximation of reality for the calibrated weights for the number of ECD enrolled children per age group in Quintiles 4 and 5

The factors of the early childhood system combine to produce *F*^*B*^, the main factor supporting child aptitude. The weights *w*^*B*^, *w*^*T*^, and *w*^*D*^ are determined through parameter calibration so that the lowest average root mean square error (RMSE) for each childhood age and its simulated values are achieved. The lowest average RMSE of 14% and 15%, is achieved when *w*^*B*^ = *w*^*T*^ = *w*^*D*^ = 1, for both Quintiles 1–3 and Quintiles 4 and 5 schools, respectively. Figures 4 and 5 contain the ECDM approximation of reality for 2010 to 2016 for all enrolled children in both Quintiles 1–3 and Quintiles 4 and 5. A perfect approximation would cause a zero deviation from the target value. The simulation for the Quintiles 1–3 system deviates from the target within the interval of [− 10%, 15%] for all ages of children simulated. The simulation for the Quintile 4 and 5 system deviates from the target within the interval of [− 5%, 10%].

The results of the ECDM are subject to three major assumptions of estimated parameters. The first is the calibrated factor weights of the early childhood system depicted in Fig. 2 and the second is some of the initial values for each factor in this system. The third is the division of the number of children enrolled into the different quintiles.

Sensitivity analysis on these values gives greater insight into areas for potential intervention. Sensitivity analysis is, therefore, only relevant for the Quintiles 1–3 system as interventions are only performed on these quintiles to compare their behavior with that of Quintiles 4 and 5.

### Factor weights within the early childhood system

**Fig. 6:**
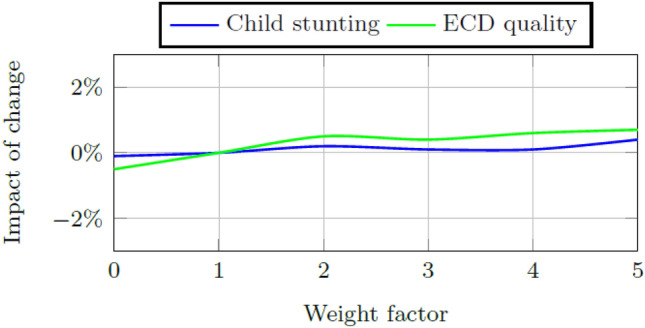
Impact on the final child aptitude score per change in the weight size of ECD quality and child stunting, respectively, in Quintiles 1–3

**Fig. 7:**
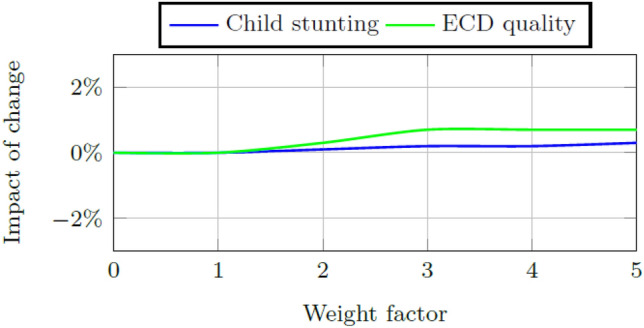
Impact on the final success measure per change in the weight size of ECD quality and child stunting, respectively, in Quintiles 4 and 5

Two indicators are used to measure the health of the system. The child aptitude score is the aggregate measure of the early childhood system’s ability to increase or decrease children’s school readiness and the success measure is the ratio of total school ready five year olds to the total number of five year olds within the system. Figures 6 and 7 show the impact of change in these indicators when the weights for ECD quality and child stunting, respectively are increased or decreased by a factor of 0.5 to a minimum of 0 and a maximum of five times the calibrated weight. Model results are affected less by changes to the weight of child stunting than by changes to the weight of the ECD quality score, but the impact of weight changes to either is small, i.e., less than 1% increase or decrease.

### Initial stock values within the early childhood system

**Fig. 8:**
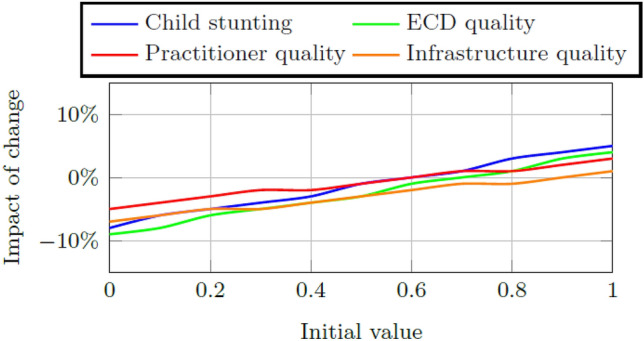
Impact on the final child aptitude score per change in the initial value of child stunting, ECD quality, practitioner quality, and infrastructure quality, respectively, in Quintiles 1–3

**Fig. 9:**
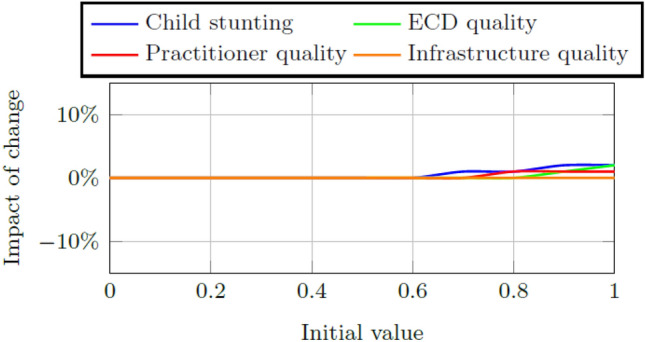
Impact on the final success measure per change in the initial value of child stunting, ECD quality, practitioner quality, and infrastructure quality, respectively, in Quintiles 4 and 5

Figures 8 and 9 contain the impact of change in these indicators when the weights for child stunting, ECD quality, practitioner quality, and infrastructure quality respectively are changed. Predictably from the structure of Fig. 2, changes to the initial values of child stunting and ECD quality have a greater impact on the final child aptitude score than changes to the practitioner and infrastructure quality, albeit to a small maximum change of approximately 5%. The child progression trains are much less sensitive to changes in these initial values with changes less than 2% observable when the values are increased to values higher than 0.6. The relatively high values (i.e., the values greater than 0.7 in Table 11) of the factors within the early childhood system absorb large decreases in the factors tested for sensitivity so that the success measure is not affected.

### Initial enrolment distribution

**Fig. 10:**
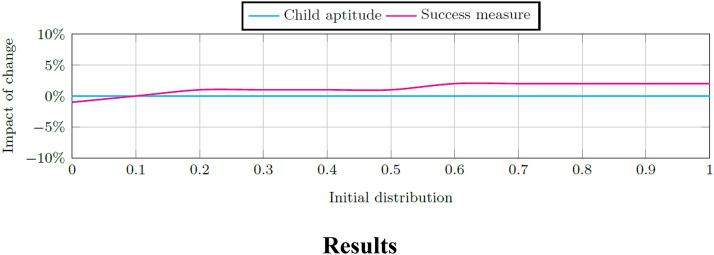
Impact on the final child aptitude and success measure per change in the initial distribution of enrolled and not-enrolled children

The model's sensitivity to changes in the initial distribution of enrolled to not-enrolled children is decreased to a minimum enrolment of 0% to a maximum of 100%. Figure 10 shows the impact of change on the child aptitude and success measure for each change to the initial population distribution. The best success measure is achieved when more than 60% of children are enrolled in ECD programs, but this increased enrolment has no impact on the system's ability to increase or decrease child aptitude.

## Results

**Fig. 11:**
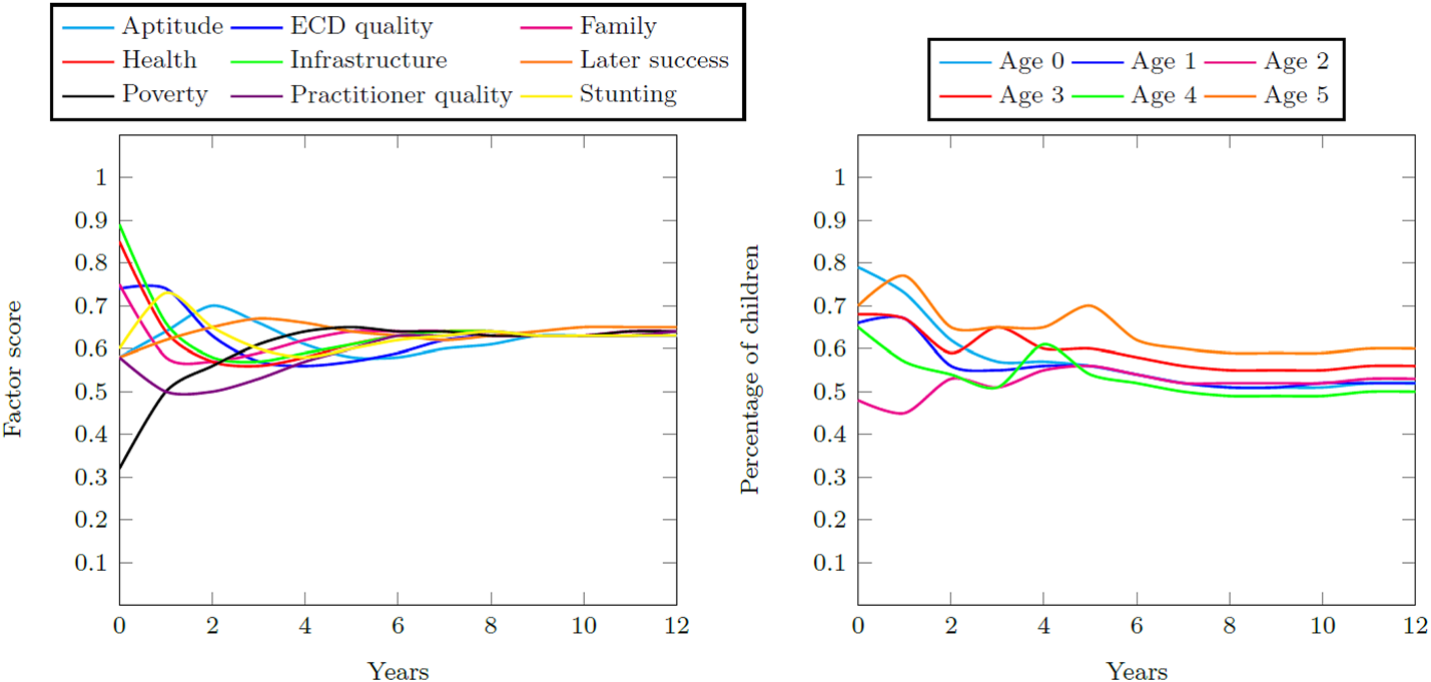
Factor scores during the 12-year simulation period and the percentage of children with adequate development for their age for the Quintiles 1–3 base case

Figure 11 contains the resultant factor scores and child distributions during the 12-year simulation period for the Quintiles 1–3 system. The lack of exogenous variables to the system causes the convergence of the factors to an equal score over time. The impact of the low poverty score visibly draws the initially high infrastructure and health scores downwards so that the system achieves a final aptitude goodness score of 0.62. This score ultimately produces a final percentage of school-ready five year olds at 60% of all children. Therefore, the success measure (i.e., the ratio of total school-ready five year olds to the total number of five year olds) is 0.60.

**Fig. 12:**
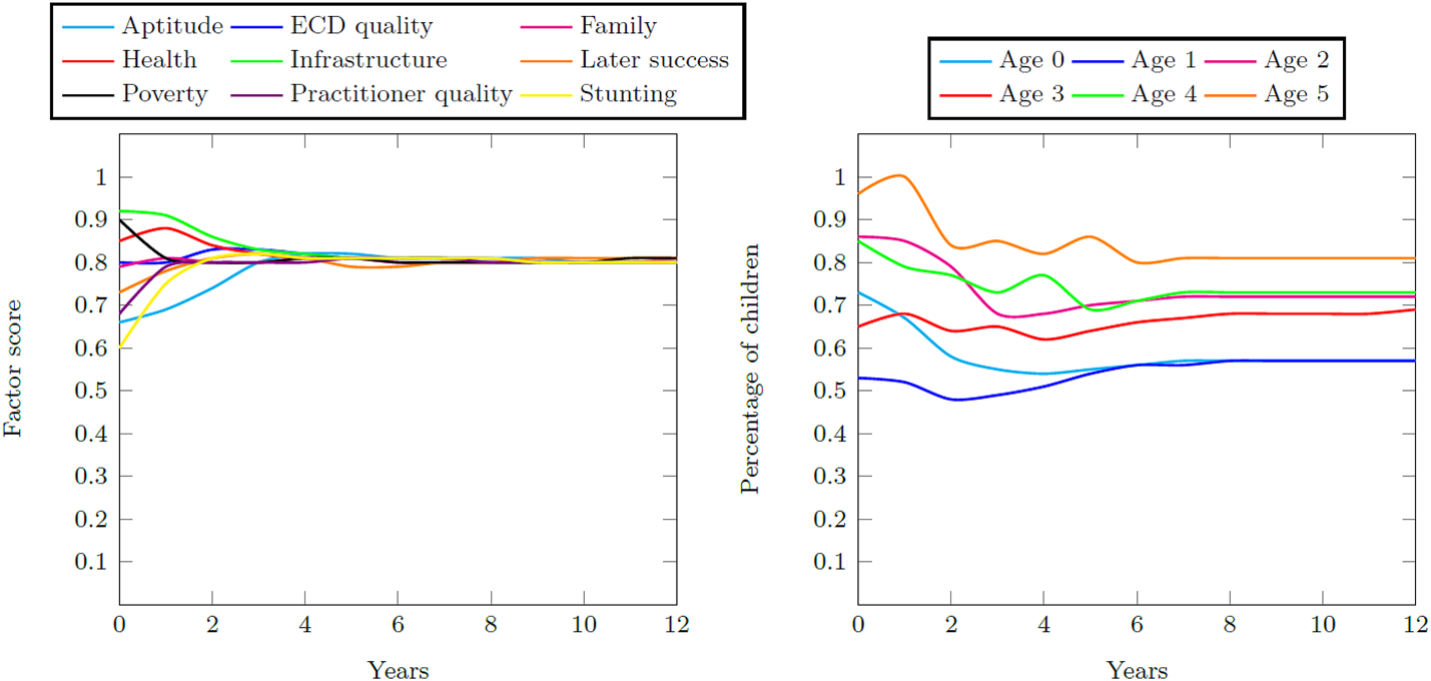
Factor scores during the 12-year simulation period for the Quintile 4 and 5 base case and the percentage of children with adequate development for their age for the Quintile 4 and 5 base case

Figure 12 contains the resultant factor scores and child distributions during the 12-year simulation period for the Quintile 4 and 5 system. The high initial values for the system factors remain high throughout the simulation period so that a final aptitude goodness score of 0.80 is achieved. This high score produces a final percentage of school-ready five year olds at 81% of all children. The success measure, therefore, is 0.81.

### Interventions

Three interventions are run to determine whether it is the number of children enrolled into EDC programs or the quality of the programs that are contributing to the low percentage of school-ready five year olds in Quintiles 1–3 communities. Venter and Vosloo (2018) show that late, once-off, and singular interventions are incapable of improving a system. Therefore, interventions are applied early, consistently, and on multiple factors to examine their impact.

### Increased ECD program quality

**Fig. 13:**
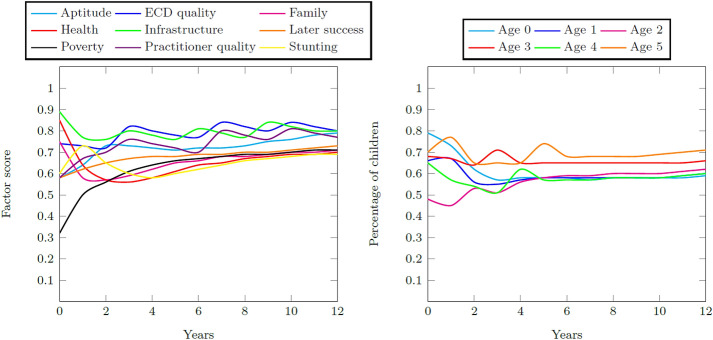
Factor scores for the quality intervention and the percentage of children with adequate development for their age for Quintiles 1–3 for the quality intervention

A large intervention where the factor score is increased by the maximum is applied to ECD quality, practitioner quality, and infrastructure quality for the Quintiles 1–3 system. A large intervention is the equivalent of ensuring that all ECD programs are operating at maximum effectiveness. Figure 13 contains the resultant factor scores and distribution of children for this intervention. Increasing practitioner quality is effective in particular as the other two factors are initially strong and need only to be maintained. The regular increase in ECD education quality has an improvement effect on the system as a whole so that a final aptitude goodness score of 0.78 is achieved. The percentage school-ready five year olds increases to 70% of all children so that the final success measure is 0.70.

### Increased ECD enrolment quantity

**Fig. 14:**
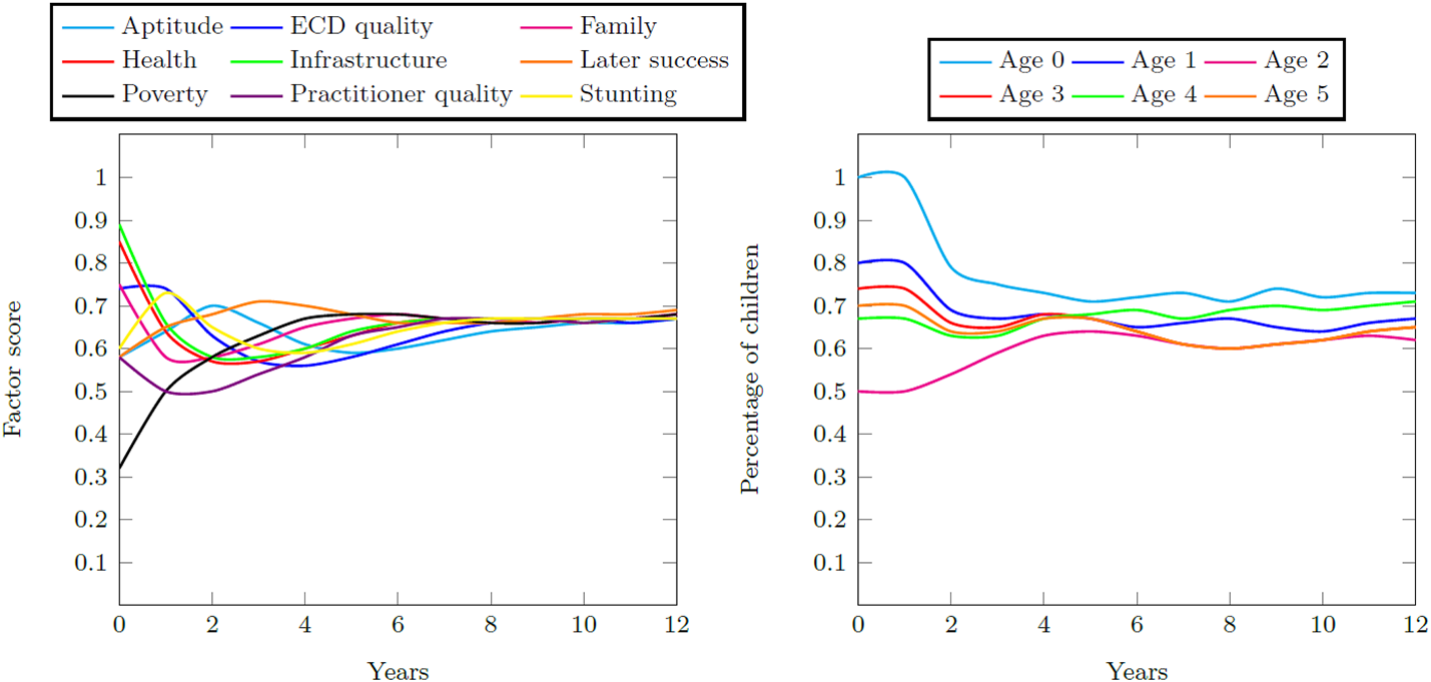
Factor scores for the increased enrolment intervention and the percentage of children with adequate development for their age for Quintiles 1–3 for the increased enrolment intervention

The ECDM is initialized so that all children are enrolled in ECD education. This means that no children grow to the age of five having been exposed to only informal or no education. Figure 14 contains the resultant factor scores and children distributions for this intervention. Exposing all children to early childhood development at its base case quality improves their cognitive development for only the two and four year olds. It cannot improve the percentage for children of other ages, but it is strong enough to maintain some school-ready five year olds at 65% of all five year olds. A final aptitude goodness score of 0.66 is achieved and the final success measure is 0.66.

### Decreased poverty

**Fig. 15:**
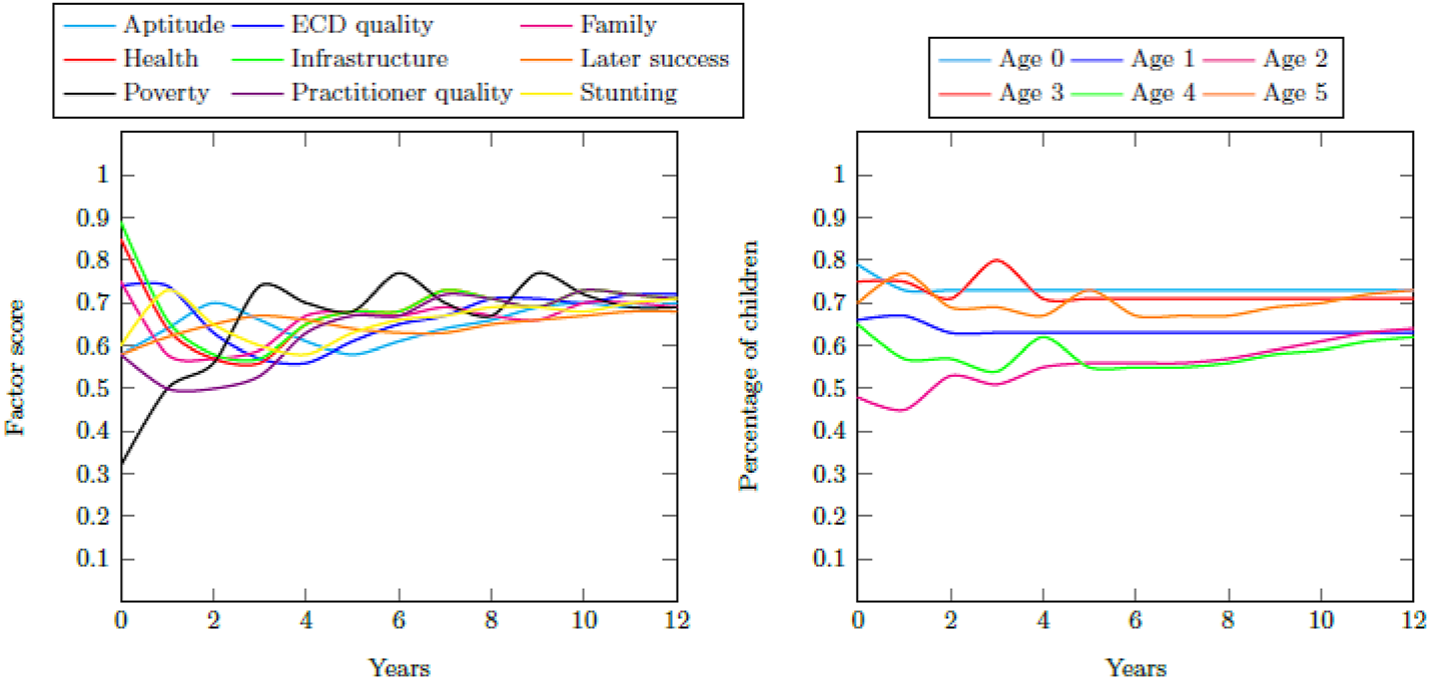
Factor scores for the decreased poverty intervention and the percentage of children with adequate development for their age for Quintiles 1–3 for the increased enrolment intervention

All four of the system’s reinforcing loops depicted in Fig. 1 include poverty as a factor. Family poverty also has the greatest difference between the initial values of the Quintiles 1–3 and Quintiles 4 and 5. It is, therefore, necessary to analyze the impact of an intervention in this area on the resultant performance measures. A large intervention is applied to poverty for the Quintiles 1–3 system, where a large intervention takes 3 years before its impact is experienced and is equivalent to providing an extreme form of regular poverty relief. Figure 15 contains the resultant factor scores and distribution of children for this intervention. The regular decrease in the poverty score has an improvement effect on the system as a whole so that a final aptitude goodness score of 0.80 is achieved. The percentage school ready five year olds increases to 71% of all children so that the final success measure is 0.70.

## Discussion

With regards to Early Childhood Development, the question arises whether the DBE should focus its efforts on increasing the enrolment rate into ECD programs, or whether it should focus its efforts instead on improving the programs into which the children are enrolled. The Early Childhood Development Model can be used to answer the question of which intervention increases the number of school-ready five year olds in the Western Cape. The model is populated with data from national data sets and the model is run for the 12 years leading up to 2030.

Initial values for the weights of the factors impacting childhood aptitude are determined through parameter calibration. Validation of this calibration results in an average RMSE of 14% and 15% for each system, respectively, when the simulated percentage of children per age group is fitted to the actual number.

A sensitivity analysis is done on the initial weights of child stunting and ECD program quality, respectively. The model is more sensitive for changes to the weight of child stunting although the resultant changes to child aptitude and the success measure are very small (i.e., smaller than 2%). Further sensitivity analysis is done on the initial stock values of child stunting, ECD program quality, practitioner quality, and infrastructure quality, respectively. Again the model is more sensitive to changes in the initial stock values of child stunting and ECD quality indicating that interventions in these areas are expected to cause the greatest impact of change on the system.

The base case results show that schools in socio-economic Quintiles 1–3 communities stabilize at a childhood aptitude score so that up to 60% of all five year olds in these communities are adequately prepared to enter Grade 1. In contrast to this, children in socio-economic Quintile 4 and 5 communities stabilize at a childhood aptitude score so that up to 81% of all five year olds in these communities are adequately prepared.

Three interventions explore the research question of whether it is the number of enrolments into ECD programs that increases a cohort’s school readiness, or rather the quality of the ECD programs into which they were enrolled. A timely, continuous, and large intervention in the quality of the ECD system for the Quintiles 1–3 system leads to a final child aptitude score increase of 0.16 and a success measure increase of 0.10. A timely, continuous, and large intervention in the percentage of children enrolled into formal ECD programs in the Quintiles 1–3 system leads to a final child aptitude increase of 0.04 and a success measure increase of 0.06. A timely, continuous, and large intervention to decrease family poverty in the Quintiles 1–3 system leads to a final child aptitude increase of 0.18 and a success measure increase of 0.10. None of the interventions enable the Quintiles 1–3 system to match the success measure of the Quintiles 4 and 5 system. Table 14 contains a summary of the results.

**Table 14:** Summary of the base case and intervention results for the ECDM

	Aptitude score	Success measure
Quintiles 1–3 base case	0.62	0.60
Quintiles 4 and 5 base case	0.80	0.81
Program quality intervention	0.78	0.70
Enrolment intervention	0.66	0.66
Poverty intervention	0.80	0.70

## Conclusions

The results answer the research question for the Western Cape by showing that increasing the quality of the formal ECD programs leads to a greater percentage of school-ready five year olds than increasing the percentage of enrolled children, but that decreasing community poverty leads to better results than either intervention. Practically this implies that interventions should focus on decreasing the unemployment rate of parents, and allocating more funds to training practitioners, and procuring resources for ECD facilities.

The structure of the ECDM allows for further research questions to be answered through its application. Interventions on any of the system factors may be compared with each other. For example, equally interesting would be a research question as to whether decreasing child stunting is a more effective intervention than improving parental level of education, or any such combination.

The Western Cape province is one of the richest and most functional provinces in the country. The conclusions listed in this paper cannot be assumed to be true for the country as a whole. Policy researchers can populate the model with province-specific data to test the research question in the context of the remaining eight provinces to get specific recommendations for the South African system. Modelers can also expand the ECDM to include the developmental stage before birth as it currently excludes differentiation of children based on their prenatal circumstances. Lack of adequate nutrition, fetal alcohol syndrome, and other factors during pregnancy can influence cognitive development and should be considered for even more accurate analysis.

The ECDM as described in this paper compares the system of socio-economic Quintiles 1–3 to that of socio-economic Quintiles 4 and 5. There are, however, many other dimensions within which to categorise communities for richer comparison. Questions of race, gender, language of education can be explored using the ECDM.

Simply recommending that a community’s level of poverty should be reduced to increase the school readiness of its children is no easy recommendation to implement practically. The model can be expanded so that experiments with a universal basic income might be performed.

The input data and results reported reflect the South African reality before the devastating impact of the COVID-19 pandemic starting in 2020. Basic education in South Africa was particularly impacted by lockdowns and regulations. The model presented could be used to re-evaluate pre-pandemic policies given updated data and new research questions. The economic damage of lockdowns, isolation, and increased parental unemployment must in future be considered as the fourth socio-economic quintile has not less in common with the richest quintile, and more in common with the poorer three quintiles.

## Data Availability

The data and materials contained within this research are public domain and available as an open source.
